# Multilingualism Among First-Year Resident Physicians

**DOI:** 10.1001/jamanetworkopen.2025.42587

**Published:** 2025-11-10

**Authors:** Pilar Ortega, Montserrat Tijerina, Rahardhika Utama, Kenji Yamazaki, Sean O. Hogan, Lisa C. Diamond, Muveddet Harris

**Affiliations:** 1 Department of Medical Education, University of Illinois College of Medicine, Chicago; 2 Department of Emergency Medicine, University of Illinois College of Medicine, Chicago; 3 Pritzker School of Medicine, University of Chicago, Chicago, Illinois; 4 Accreditation Council for Graduate Medical Education, Chicago, Illinois; 5 Immigrant Health and Cancer Disparities Service, Hospital Medicine Service, Memorial Sloan Kettering Cancer Center, New York, New York

## Abstract

This cohort study characterizes the non–English language proficiency of postgraduate year-1 resident physicians and compares resident physicians languages with population language needs.

## Introduction

When clinicians and patients communicate directly in a shared language (termed language-concordant care), health outcomes and satisfaction improve as costs decrease.^1^ More than 68 million US persons speak a non-English language at home.^2^ Despite legal and Joint Commission standards, physicians often resort to limited language skills and underuse interpreters. Clinical learning environments may be underequipped to identify the language skills of trainees and provide accessible resources to ensure safe patient care in non-English languages.^3^

The aims of this study were to characterize the language skills of postgraduate year-1 (PGY1) resident physicians, explore factors associated with advanced multilingualism, and compare PGY1 resident physician languages with population language needs.

## Methods

We conducted a retrospective cohort study, analyzing the languages and proficiency levels of physicians completing their first year of residency between 2022 and 2024. We included resident physicians with an Association of American Medical Colleges ID and Electronic Residency Application System (ERAS) language data and who were in the Accreditation Council for Graduate Medical Education (ACGME) database (eMethods in Supplement 1). This study was deemed exempt by the American Institutes for Research Institutional Review Board as secondary analysis of existing data and followed the STROBE reporting guideline.

The primary outcome was self-assessed language proficiency^4^ from the ERAS application (eTable in Supplement 1). Secondary variables included heritage language exposure (non-English language use in childhood homes), race and ethnicity, and medical school type (eTable in Supplement 1). Race and ethnicity are reported as given in the ACGME database.

For each of the top 40 languages reported by the American Community Survey,^2^ we calculated the number of PGY1 resident physicians with advanced or native skills per 100 000 US persons with limited English proficiency (LEP). Data were analyzed using R version 4.5.0 (R Foundation for Statistical Computing). A 2-sided *P* < .05 was considered statistically significant.

## Results

Of 116 552 PGY1 resident physicians, 107 610 had sufficient data to be included. Of these, 77 862 (72.3%) reported non-English language skills at any proficiency level. Across PGY1 resident physicians, 40 286 (37.4%) reported advanced or native and 37 576 (34.9%) indicated novice or intermediate multilingualism (Table). Compared with White PGY1 resident physicians, Asian residents had higher odds of advanced or native multilingualism (odds ratio [OR], 8.5 [95% CI, 8.2-8.8]; *P* < .001); as did Black or African American residents (OR, 1.9 [95% CI, 1.8-2.0]; *P* < .001); residents who identify as Hispanic, Latino, or of Spanish origin (OR, 11.3 [95% CI, 10.8-11.8]; *P* < .001); and Native Hawaiian or Other Pacific Islander residents (OR, 4.5 [95% CI, 3.5-5.8]; *P* < .001).

**Table.** Language Proficiency and Heritage Exposure by Race and Ethnicity and Medical School Type for First-Year Resident Physicians From 2022-2024

Variable	PGY1 resident physicians, No./total No. (%)					
	By language proficiency (n = 107 610)^a^			By heritage language exposure (n = 80 972)^b^		
	English only (n = 29 748)	Multilingual		English only (n = 20 164)	Multilingual	
		Novice or intermediate (n = 37 576)	Advanced or native (n = 40 286)		Nonheritage learner (n = 32 597)	Heritage learner (n = 28 211)
** Race and ethnicity^c^**						
American Indian or Alaska Native	84/124 (67.7)	35/124 (28.2)	5/124 (4.0)	59/111 (53.2)	40/111 (36.0)	12/111 (10.8)
Asian	1934/30 023 (6.4)	9290/30 023 (30.9)	18 799/30 023 (62.6)	1212/19 598 (6.2)	2963/19 598 (15.1)	15 423/19 598 (78.7)
Black or African American	3169/7737 (41.0)	2473/7737 (32.0)	2095/7737 (27.1)	1987/5303 (37.5)	1742/5303 (32.8)	1574/5303 (29.7)
Hispanic, Latino, or of Spanish origin	860/11 199 (7.7)	2606/11 199 (23.3)	7733/11 199 (69.1)	623/7803 (8.0)	2173 (27.8)	5007/7803 (64.2)
Multiple race/ethnicity	1520/5294 (28.7)	2273/5294 (42.9)	1501/5294 (28.4)	1047/4252 (24.6)	2053/4252 (48.3)	1152/4252 (27.1)
Native Hawaiian or Other Pacific Islander	38/232 (16.4)	85/232 (36.6)	109/232 (47.0)	22/159 (13.8)	59/159 (37.1)	78/159 (49.1)
Unknown^d^	166/2446 (6.8)	576/2446 (23.5)	1704/2446 (69.7)	90/1375 (6.5)	214/1375 (15.6)	1071/1375 (77.9)
White	21 977/50 555 (43.5)	20 238/50 555 (40.0)	8340/50 555 (16.5)	15 124/42 371 (35.7)	23 353/42 371 (55.1)	3894/42 371 (9.2)
**Medical school type**						
Canadian	3/38 (7.9)	9/38 (23.7)	26/38 (68.4)	2/15 (13.3)	7/15 (46.7)	6/15 (40.0)
International^e^	2219/22 270 (10.0)	3154/22 270 (14.2)	16 897/22 270 (75.9)	857/4303 (19.9)	1243/4303 (28.9)	2203/4303 (51.2)
US allopathic	20 074/64 647 (31.1)	26 570/64 647 (41.1)	18 003/64 647 (27.8)	14 829/61 403 (24.2)	25 719/61 403 (41.9)	20 855/61 403 (34.0)
US osteopathic	7452/20 655 (36.1)	7843/20 655 (38.0)	5360/20 655 (26.0)	4476/15 251 (29.3)	5628/15 251 (36.9)	5147/15 251 (33.7)
**Heritage language exposure^b^**						
Multilingual heritage learner	NA	8149/28 211 (28.9)	19 202/28 211 (68.1)	NA	NA	NA
Multilingual nonheritage learner	NA	20 902/32 597 (64.1)	3526/32 597 (10.8)	NA	NA	NA

Abbreviations: NA, not applicable; PGY1,postgraduate year-1.

^a^ Language proficiency categorizations include advanced or native multilingual, denoting PGY1 resident physicians who reported an advanced or native or functionally native level on any non-English language in the Electronic Residency Application System; novice or intermediate multilingual, denoting those who reported basic, fair, or good; and English only, those who did not report skills in any non-English language.

^b^ Heritage language exposure categorizations include multilingual heritage learner, denoting individuals who reported speaking a non-English language and indicated always or often for language use in childhood home in the American Medical College Application Service; multilingual nonheritage learner, denoting those who speak a non-English language and were exposed to it from time to time, rarely, or never in their childhood home; and English only, those who did not report skills in any non-English language.

^c^ Race and ethnicity reporting follows the Integrated Postsecondary Education Data System guidance: Individuals who report Hispanic and any other race or ethnicity are reported as Hispanic. Individuals who report any other 2 race or ethnicity categories are reported as multiple race or ethnicity.

^d^ The unknown category includes responses of undefined, unknown, other, and prefer not to report, and those who did not answer the question.

^e^ International medical school was defined as a medical school outside of the US or Canada.

Of the 80 972 (75.2%) PGY1 resident physicians with heritage language data, 94.7% (76 654) were US medical graduates and 34.8% (28 211) reported frequent non-English language childhood exposure (termed heritage learners) (Table). Heritage learners had higher odds of advanced or native proficiency compared with nonheritage learners (OR, 13.9 [95% CI, 13.3-14.6]; *P* < .001).

For every 100 000 US persons with LEP, 182 PGY1 resident physicians reported advanced or native skills across all non-English languages (Figure). Four of the top 5 population languages (Spanish, Tagalog, Vietnamese, and Chinese) had among the lowest ratios of language-concordant physicians.

**Figure.**
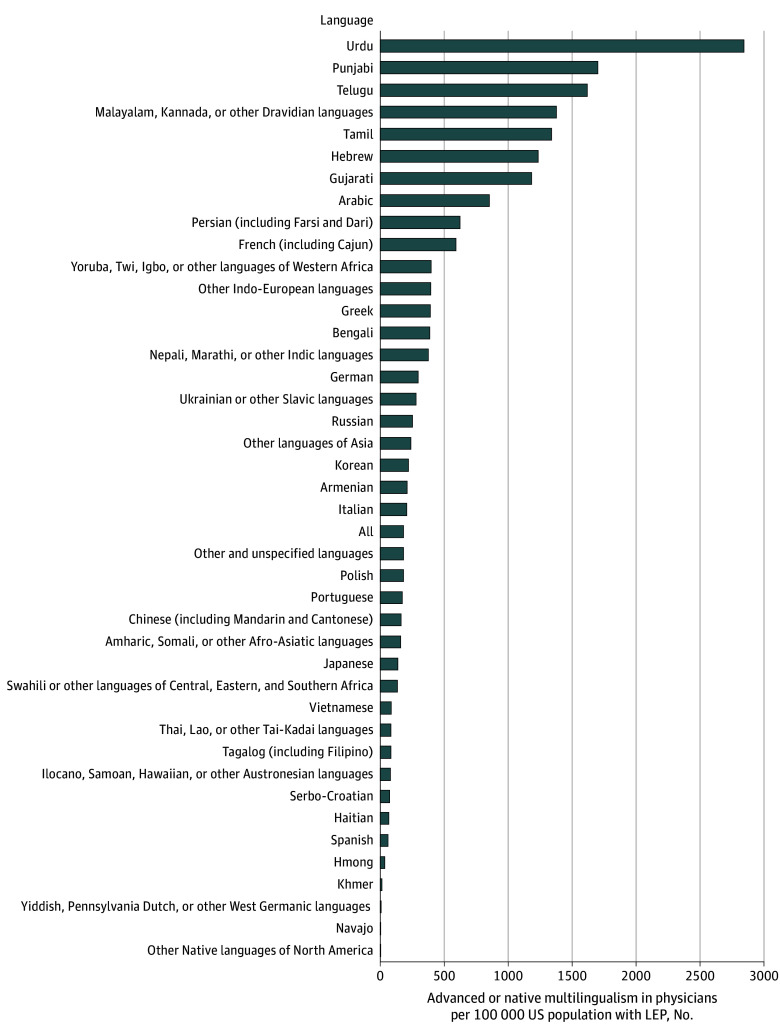
Languages Spoken With Advanced or Native Proficiency by First-Year Resident Physicians From 2022-2024 Relative to US Population Need US population data are an average of yearly estimates for 2021-2023 from the American Community Survey, which reports the number of persons aged 5 years and over who speak English less than “very well” and are thus categorized as having limited English proficiency (LEP). The term *all* denotes all languages represented by persons who indicated speaking a non-English language at home and were categorized as LEP; in the study time frame, there were 182 postgraduate year-1 resident physicians with at least advanced proficiency across all reported non-English languages for every 100 000 persons with LEP.

## Discussion

Multilingualism is common among resident physicians. The prevalence of PGY1 advanced or native-level skills in our study (37.4%) contrasts with 49.7% previously reported among 2013 ERAS applicants.^5^ This difference may point to a more linguistically diverse population of applicants compared with those who successfully match.

More than two-thirds of heritage learners reported advanced or native proficiency compared with one-tenth of nonheritage learners. This finding supports prior work^6^ indicating that heritage exposure is associated with, but not a proxy for, advanced proficiency. Heritage learners, who often have valuable cultural ties to the language and community, should not be assumed to be ready for language-concordant care without training and testing.^6^

Limitations include lack of language data from sources outside of ERAS and American Medical College Application Service, potentially underestimating international and Canadian medical school graduates who did not complete those applications. Also, the available data did not capture signed languages and did not disaggregate certain language groupings (eg, Chinese).

Understanding the self-assessed language skills of medical student, resident, and faculty applicants may help institutions tailor language resources—such as interpreters, education, and testing^6^—to learner and clinician proficiency levels for locally prevalent languages. Future research should evaluate patient outcomes and language-specific population needs.

## Notes

Supplement 1. **eMethods.** Additional Information on Data Set Creation and Analysis **eTable.** Key Variables

Supplement 2.
Data Sharing Statement
