# Supplementary material for: Multilingualism Among First-Year Resident Physicians
Source: JAMA Netw Open. 2025 Nov 10;8(11):e2542587. doi: 10.1001/jamanetworkopen.2025.42587 (PMC12603852; doi:10.1001/jamanetworkopen.2025.42587)
Supplement: Supplement 1. — eMethods. Additional Information on Data Set Creation and Analysis eTable. Key Variables [file jamanetwopen-e2542587-s001.pdf]

Supplemental Online Content

Ortega P, Tijerina M, Utama R, et al. Multilingualism among first-year resident physicians. *JAMA Netw Open*. 2025;8(11):e2542587. doi:10.1001/jamanetworkopen.2025.42587

eMethods. Additional Information on Data Set Creation and Analysis
eTable. Key Variables

This supplemental material has been provided by the authors to give readers additional information about their work.

© 2025 Ortega P et al. *JAMA Network Open*.

eMethods. Additional Information on Data Set Creation and Analysis

To construct the dataset, we first identified 116,552 PGY1s in the Accreditation Council for Graduate Medical Education (ACGME)'s database for those three academic years. Of those PGY1s, 3,267 (2.8%) were excluded due to not having an Association of American Medical Colleges (AAMC) ID (a unique numerical identifier needed to merge Electronic Residency Application Services (ERAS) language data with ACGME demographic data), and 5,675 (4.9%) were excluded due to lack of ERAS language data. Physicians who did not have an AAMC profile, such as those who did not complete an American Medical College Application Service (AMCAS) or ERAS application, may not have an AAMC ID and would not have language proficiency information in our data set. The final dataset consisted of 107,610 PGY1s (92.3%).

Heritage language exposure data were only available for PGY1s who applied to allopathic medical schools through the AMCAS application, which primarily consisted of US medical graduates. Hence, heritage language data were not available for PGY1s who did not complete an AMCAS application, such as those who only applied to osteopathic and/or non-US medical schools. A small number of international medical graduates (IMGs) and Canadian medical school graduates are included in the heritage language data, representing individuals who *applied* to US allopathic medical schools but completed medical school outside of the US. Therefore, our study's heritage language exposure analysis primarily provides insights on the language acquisition of US medical school graduates. While prior research has shown that the majority of US IMGs speak a language besides English by acquiring the dominant language of their country of origin, the language acquisition patterns of US medical school graduates have not been well-studied. After excluding those for whom AMCAS data were unavailable, the heritage exposure dataset included 80,972 PGY1s (75.2% of 107,610).

We used descriptive statistics to summarize frequency and calculate percentages. We used logistic regression to calculate the odds of reporting advanced/native multilingualism by race/ethnicity categories compared to PGY1s who identified as White (R version 4.5.0, Binomial GLM). We considered a two-sided P value <.05 to be statistically significant.

© 2025 Ortega P et al. *JAMA Network Open*.

eTable. Key Variables

Key Variable	Description	Data Source
Language proficiency	During the years of this study, AAMC's ERAS applicants were asked to report the languages they speak and self-assess their level in each language on a 5-point scale (native/functionally native, advanced, good, fair, and basic). The ERAS language proficiency scale was modified from the validated Interagency Language Roundtable scale for Healthcare (ILR-H).[1] Both scales maintain the same 5 proficiency levels, with minor wording changes made on the ERAS scale for clarity and conciseness.[2] To meaningfully dichotomize the data, we grouped PGY1s into an "advanced/native multilingual"* category if they reported speaking any non-English language at an "advanced" or "native/functionally native" level, the ILR-H levels that correspond with more than two-thirds pass rate on a formal proficiency test.[3] We grouped PGY1s into a "novice/intermediate multilingual" category if they indicated "basic," "fair," or "good." If PGY1s indicated speaking more than one non-English language, we included the proficiency level of the language for which they reported the highest level. We characterized PGY1s as "English only" if they did not report any language besides English.	ERAS, Self-reported
Heritage language exposure	The AMCAS application asked applicants to report on their childhood exposures to non-English languages. We defined "heritage learners" as individuals who reported "always" or "often" to frequency of language use in childhood homes, consistent with previously published measures of heritage exposure.[4] We defined "non-heritage learners" as individuals who reported a non-English language but described language use at home "from time to time," "rarely," or "never."	AMCAS, Self-reported
Race/ethnicity and gender	We investigated associations with race and ethnicity because prior research shows that residency applicants with certain racial or ethnic backgrounds have a higher likelihood of reporting a language other than English. We sought to explore whether these associations were also true for applicants who successfully matched in residency.[5] Race and ethnicity categories followed the Integrated Postsecondary Education Data System standards: Individuals who identified as Hispanic, Latino,	ACGME database

© 2025 Ortega P et al. *JAMA Network Open*.

	or of Spanish origin (hereafter, Hispanic) plus any other race/ethnicity were categorized as Hispanic. Those who were non-Hispanic and reported more than one race/ethnicity were classified as “Multiple race or ethnicity.” The ACGME populates their resident database using self-reported race/ethnicity and gender data whenever possible; if unavailable, data are supplemented from program-reported data submitted by the resident’s graduate medical education program to the ACGME.	
Medical school type	We categorized individuals as coming from US allopathic, US osteopathic, international (outside of the US), and Canadian medical schools. We categorized Canadian medical schools and US medical schools separately due to differences in the linguistic context between Canada and the US that may affect the language profile of medical school graduates.	ACGME database

Abbreviations: AAMC, Association of American Medical Colleges; ACGME, Accreditation Council for Graduate Medical Education; AMCAS, American Medical College Application Service; ERAS, Electronic Residency Application Service; ILR-H, Interagency Language Roundtable scale for Healthcare; PGY1, post-graduate year 1 or first-year resident physician.
*Multilingualism is defined as speaking English and at least one other language.

eTable References

1. Diamond LC, Luft HS, Chung S, Jacobs EA. "Does this doctor speak my language?" Improving the characterization of physician non-English language skills. Health Serv Res. 2012;47(1 Pt 2):556-569. doi:10.1111/j.1475-6773.2011.01338.x.
2. Ortega P, McDade W. Language proficiency: how the Physician Data Initiative’s data collection and reporting standard impacts graduate medical education. J Grad Med Educ. 2025;17(2):254–259. doi:10.4300/JGME-D-25-00138.1
3. Diamond L, Toro Bejarano M, Chung S, et al. Factors associated with accuracy of self-assessment compared with tested non-English language proficiency among primary care providers. Med Care. 2019;57(5):385-390. doi:10.1097/MLR.0000000000001105.
4. Ortega P, Figueroa JA, Gregorich SE, et al. An Exploratory Investigation of Heritage and Educational Language Exposures as Factors in Medical Student Spanish Language Proficiency. J Gen Intern Med. Published online January 22, 2025. doi:10.1007/s11606-025-09357-1
5. Diamond L, Grbic D, Genoff M, et al. Non-English-language proficiency of applicants to US residency programs. JAMA. 2014;312(22):2405-2407. doi:10.1001/jama.2014.15444

© 2025 Ortega P et al. *JAMA Network Open*.
