# Supplementary material for: Multilingualism Among First-Year Resident Physicians
Source: JAMA Netw Open. 2025 Nov 10;8(11):e2542587. doi: 10.1001/jamanetworkopen.2025.42587 (PMC12603852; doi:10.1001/jamanetworkopen.2025.42587)
Supplement: Supplement 2. — Data Sharing Statement [file jamanetwopen-e2542587-s002.pdf]

Data Sharing Statement

Ortega. Multilingualism Among First-Year Resident Physicians. *JAMA Netw Open*. Published November 10, 2025. doi:10.1001/jamanetworkopen.2025.42587

Data
Data available: No

Additional Information
Explanation for why data not available: The data used for this study is part of a data-sharing agreement between the Association of American Medical Colleges and the Accreditation Council for Graduate Medical Education. Data are made available in aggregate form within the manuscript, table, and figures. Individual-level data cannot be made publicly available to protect the privacy of medical trainees.
